# Monitoring Patient Respiratory Effort During Mechanical Ventilation: Lung and Diaphragm-Protective Ventilation

**DOI:** 10.1186/s13054-020-2777-y

**Published:** 2020-03-24

**Authors:** Michele Bertoni, Savino Spadaro, Ewan C. Goligher

**Affiliations:** 1 Department of Anesthesia, Critical Care and Emergency, Spedali Civili University Hospital, Brescia, Italy; 2 Department of Morphology, Surgery and Experimental Medicine, Intensive Care Unit, University of Ferrara, Sant’Anna Hospital, Ferrara, Italy; 3 Interdepartmental Division of Critical Care Medicine, University of Toronto, Toronto, Canada; 4 Division of Respirology, Department of Medicine, University Health Network, Toronto, Canada; 5 Toronto General Hospital Research Institute, Toronto, Canada

## Abstract

This article is one of ten reviews selected from the Annual Update in Intensive Care and Emergency Medicine 2020. Other selected articles can be found online at https://www.biomedcentral.com/collections/annualupdate2020. Further information about the Annual Update in Intensive Care and Emergency Medicine is available from http://www.springer.com/series/8901.

## Introduction

At some point during mechanical ventilation, spontaneous breathing must commence. Spontaneous breathing presents a clinically important risk of injury to the lung and diaphragm. While clinicians are primarily focused on monitoring lung function to prevent ventilator-induced lung injury (VILI) during passive mechanical ventilation, less attention may be paid to the risk of VILI during assisted mechanical ventilation. Vigorous spontaneous inspiratory effort can cause both lung injury (patient self-inflicted lung injury [P-SILI]) [1, 2] and diaphragm injury (myotrauma) [3, 4]. These injuries lead to prolonged ventilation, difficult weaning, and increased morbidity and mortality [5–7]. Safe spontaneous breathing presents a complex challenge because one must aim to minimize the volume and transpulmonary pressure (*P*_L_) to avoid P-SILI while also maintaining an appropriate level of patient respiratory effort to avoid diaphragm atrophy. To this end, respiratory monitoring is key. Several practical methods are available for monitoring patient respiratory effort during assisted mechanical ventilation; this review describes their use in clinical practice.

## Mechanics of Spontaneous Breathing

**Fig. 1:**
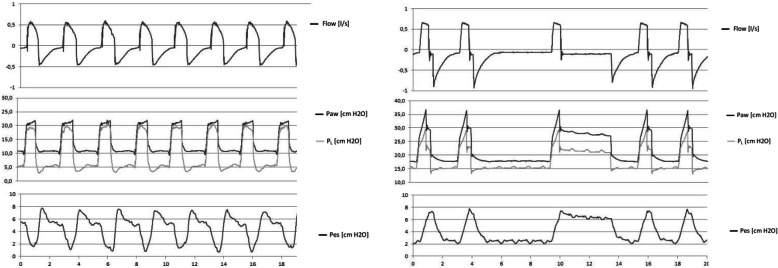
Transpulmonary pressure (*P*_L_) is generated differently in passive mechanical ventilation (upper panel) and assisted mechanical ventilation (lower panel). During passive ventilation, the pleural pressure swing is positive and transpulmonary pressure is therefore lower than airway pressure (*P*_aw_). During assisted ventilation a vigorous inspiratory effort generates a negative swing in pleural pressure resulting in an additive increase in transpulmonary pressure; transpulmonary pressure may therefore be much higher than airway pressure. *P*_*es*_ esophageal pressure

During assisted mechanical ventilation, each breath results from a negative deflection in pleural pressure (*P*_pl_) (arising from patient respiratory effort) combined with a positive airway pressure (*P*_aw_) delivered by the ventilator. The *P*_aw_ increases to the support level set on the ventilator, whereas *P*_pl_ deflects proportionally to patient effort. *P*_L_ corresponds to the difference between *P*_aw_ and *P*_pl_ (*P*_L_ = *P*_aw_ − *P*_pl_); this pressure reflects the stress applied to the lung by the combined effects of ventilator and patient effort. Although in passive mechanical ventilation *P*_aw_ is a reasonable surrogate for *P*_L_ [8], during assisted mechanical ventilation, vigorous inspiratory efforts can increase the *P*_L_ above a “safe limit.” Such excessive pressures are “unseen” when relying on the ventilator *P*_aw_ waveform; at the same airway pressure value, transpulmonary pressure could be much higher in assisted than in controlled mechanical ventilation (Fig. 1).

## Lung Injury During Spontaneous Breathing: Patient Self-Inflicted Lung Injury

**Fig. 2:**
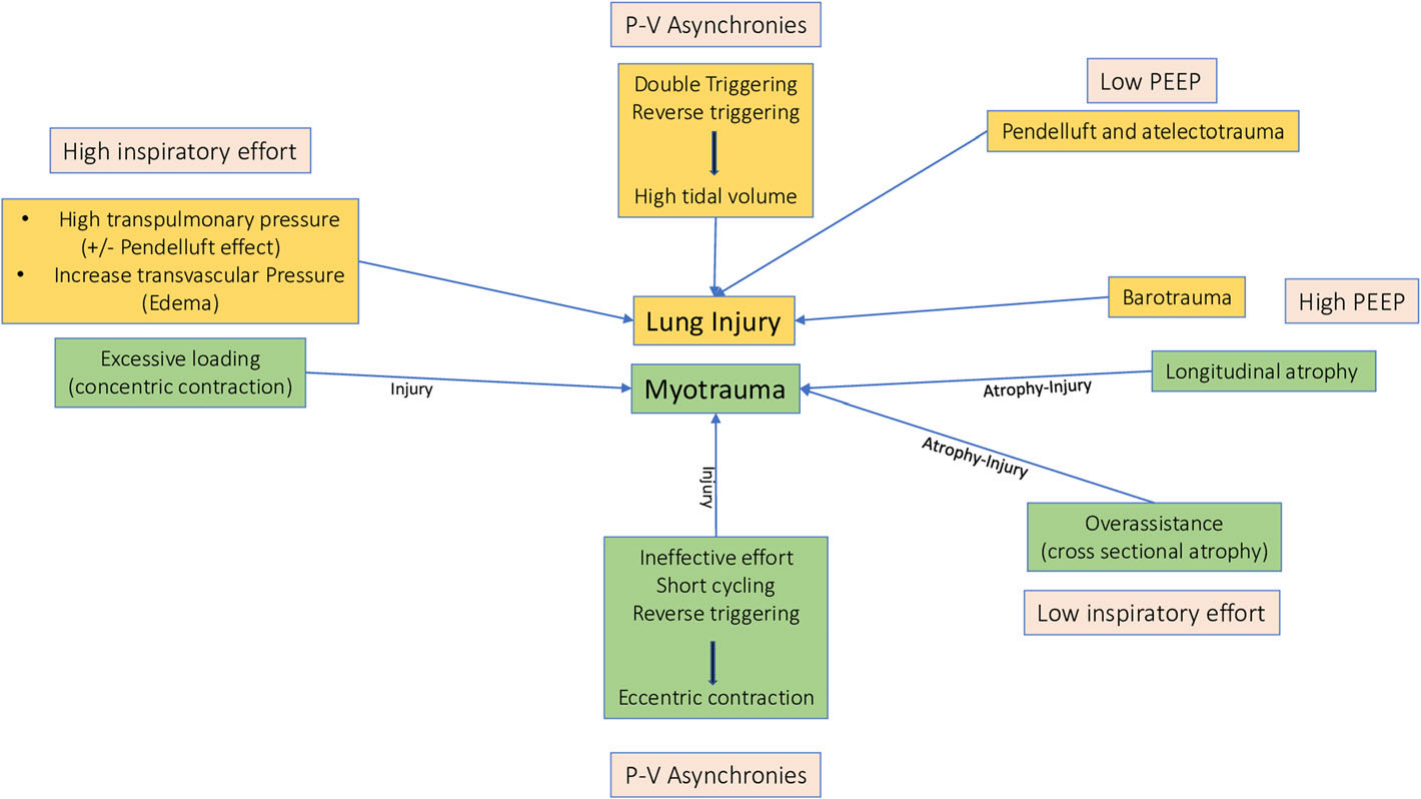
Mechanisms of lung-diaphragm injury in spontaneous breathing patients under assisted mechanical ventilation. Note that some of these mechanisms also apply under controlled mechanical ventilation (e.g., reverse triggering). *PEEP* positive end-expiratory pressure

During spontaneous breathing, vigorous patient respiratory efforts can cause lung injury (P-SILI) through different mechanisms (Fig. 2).

- *Excessive global lung stress.* As already discussed, patient respiratory efforts can increase tidal volume and *P*_L_ above safe limits when respiratory drive is elevated.
- *Excessive regional lung stress.* In the injured lung, collapsed and consolidated lung introduces parenchymal mechanical heterogeneities [9], increasing the risk of volutrauma through regional stress amplification. Mechanical stress and strain is not evenly redistributed during inflation. Consequently, inspiratory efforts generate large *P*_L_ swings in dorsal consolidated regions, resulting in the movement of air from nondependent to dependent regions (pendelluft). While this recruits collapsed lung and improves ventilation-perfusion mismatch, this phenomenon increases the overstretch of dependent lung area. In this case, the rise in *P*_L_ detected by esophageal manometry may not be a reliable measure of the local stress [10].
- *Transvascular pressure and pulmonary edema.* During spontaneous breathing, the negative *P*_pl_ generated by respiratory effort raises transvascular pressure (the pressure gradient driving fluid migration across pulmonary vessels), increasing total lung water and pulmonary edema [9, 10] and further impairing respiratory function.
- *Asynchronies.* Ventilator asynchronies, including double triggering (double mechanical breaths from a single inspiratory effort) and reverse triggering (diaphragm contractions induced by passive thoracic insufflation in passively ventilated patients) [11] can increase tidal volume and *P*_L_ and generate pendelluft, leading to lung injury.

Close monitoring of patient respiratory effort during assisted mechanical ventilation to detect and mitigate these potential injury mechanisms is therefore imperative.

## Diaphragm Injury During Spontaneous Breathing: Myotrauma

The inappropriate use of mechanical ventilation can injure not only the lung (barotrauma and volutrauma) but also respiratory muscles (myotrauma)*.* Mechanical ventilation causes myotrauma by various mechanisms, leading to a final common pathway of VIDD [5].

Mechanisms of myotrauma are summarized in Fig. 2:

- *Excessive unloading.* Over-assistance from mechanical ventilation and suppression of respiratory drive from sedation leads to acute disuse atrophy and diaphragm weakness [12]. Diaphragmatic unloading caused by over-assisted ventilation (both in control or assisted mode) is frequent during mechanical ventilation, in particular during the first 48 h. Of note, the low level of respiratory effort required to trigger the ventilator is not sufficient to avoid disuse atrophy [3], such that diaphragm atrophy can occur under pressure support ventilation.
- *Excessive concentric loading.* The diaphragm is sensitive to excessive respiratory load. Higher inspiratory patient effort, dyssynchronies, and under-assistance due to an insufficient level of support are frequent in assisted mechanical ventilation. Vigorous concentric contractions provoke high muscular tension resulting in muscle inflammation, proteolysis, myofibrillar damage, and sarcolemma disarray [13, 14]. In critically ill patients, systemic inflammation renders muscle myofibrils more vulnerable to mechanical injury ([10, 15].
- *Eccentric loading.* Eccentric contractions occur when a muscle generates contractile tension while it is lengthening (rather than shortening); such contractions are much more injurious than concentric (shortening) contractions [16]. When a low positive end-expiratory pressure (PEEP) and excessive reduction in end-expiratory lung volume are present, the diaphragm contracts even as it lengthens during the expiratory (“post-inspiratory”) phase to avoid atelectasis (“expiratory braking” phenomenon) [17]. Specific forms of dyssynchrony (reverse triggering, short cycling, ineffective effort) can generate eccentric contractions because the diaphragm is activated during the expiratory phase.
- *Excessive PEEP*. Preliminary experimental evidence suggests that maintaining the diaphragm at a shorter length with the use of excessive PEEP may cause sarcomeres to “drop out” of the muscle and shorten its length (longitudinal atrophy) [18]. This could theoretically disadvantage the length-tension characteristics of the muscle once PEEP is reduced, impairing diaphragm performance.

The first three of these injury mechanisms can be detected by monitoring respiratory effort, emphasizing the potential for such monitoring to help clinicians ensure safe spontaneous breathing during mechanical ventilation. We now proceed to review a range of monitoring techniques to achieve this goal.

## Monitoring Spontaneous Breathing Using Esophageal Pressure

The use of esophageal pressure (*P*_es_) monitoring is well-described in patients with acute respiratory distress syndrome (ARDS) under passive mechanical ventilation [19]. This technique is also the gold standard to assess respiratory effort and work of breathing but its use remains uncommon, perhaps because the utility of the information derived from *P*_es_ has been under-appreciated. When used to monitor the safety of spontaneous breathing, *P*_es_ monitoring permits several different relevant quantities to be estimated.

### Transpulmonary Pressure

*P*_es_ can be used as a surrogate measure of *P*_pl_, bearing in mind regional variations [20]. It can therefore be used to measure *P*_L_ (*P*_aw_ − *P*_pl_), by substituting *P*_pl_ with *P*_es_. As shown in Fig. 1, *P*_L_ can easily reach an injuriously high value during assisted mechanical ventilation (where both patient and ventilator distend the lung). An acceptable upper limit for *P*_L_ has not yet been defined; a “precautionary” peak inspiratory value of 20 cmH_2_O in a lung-injured patient is a reasonable target to limit the risk of injury [2, 21].

Of note, in the presence of regional ventilation heterogeneity and pendelluft, the measured value of *P*_L_ will underestimate lung stress in the dependent lung areas. While the quasi-static plateau *P*_L_ obtained during an end-inspiratory occlusion reflects lung stress during passive ventilation, the dynamic swing in *P*_L_ (Δ*P*_L_) may perhaps be more reflective of injury risk during spontaneous breathing because of the pendelluft phenomenon [22]. Δ*P*_L_ likely reflects the upper limit of mechanical stress experienced in dorsal regions of the lung under dynamic conditions [23]. Moreover, various lines of evidence suggest that the dynamic (tidal increase) in lung stress is a more important driver of lung injury than the global (peak) lung stress [24–26].

### Respiratory Muscle Pressure

**Fig. 3:**
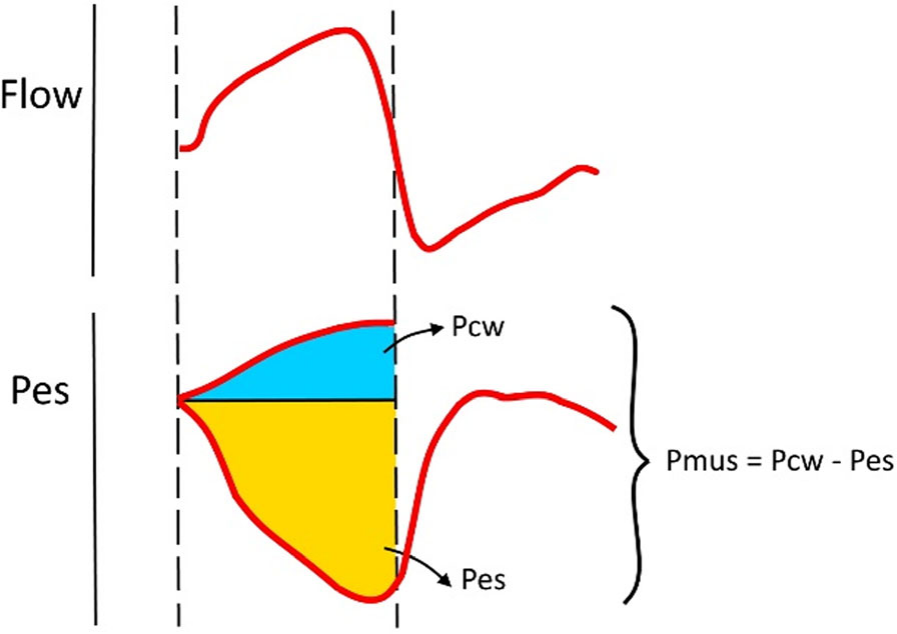
Computing inspiratory muscle pressure (*P*_mus_) from the esophageal pressure (*P*_es_) swing. *P*_mus_ derives from the difference between *P*_es_ and the added muscle pressure generated to overcome the chest wall elastic recoil (*P*_cw_). *P*_cw_ represents the elastic recoil of relaxed chest wall; it can be computed as the product of tidal volume and chest wall elastance (*E*_cw_). The *P*_mus_ area over time constitutes the pressure-time product (PTP) (yellow and blue area together)

*P*_es_ permits measurement of inspiratory effort. The inspiratory muscle pressure (*P*_mus_) corresponds to the global force generated by the inspiratory muscles. Although the diaphragm is the most important respiratory muscle, accessory inspiratory muscles (rib cage, sternomastoid, and scalene muscles) contribute significantly during vigorous effort, especially when diaphragm function is impaired. As shown in Fig. 3, *P*_mus_ is computed from the difference between *P*_es_ and the additional pressure required to overcome the chest wall elastic recoil (*P*_cw_) (*P*_mus_ = *P*_cw_ − *P*_es_).

Optimal levels of *P*_mus_ during assisted mechanical ventilation are uncertain; recent data suggest that *P*_mus_ values similar to those of healthy subjects breathing at rest may be safe and may prevent diaphragm atrophy (5–10 cmH_2_O) [4, 27]. In routine clinical practice, one can generally disregard the correction for *P*_cw_ because chest wall elastance is usually relatively low (even when pleural pressures are elevated). Hence, a target Δ*P*_es_ of around 3–8 cmH_2_O can be considered reasonably comparable to a normal *P*_mus_ of 5–10 cmH_2_O.

The gold standard measurement of respiratory effort is the integral of *P*_mus_ over the duration of inspiration (pressure-time product [PTP]) (Fig. 3). PTP is closely correlated to inspiratory muscle energy expenditure. PTP values between 50 and 100 cmH_2_O/s/min probably reflect appropriate oxygen consumption and acceptable respiratory effort [28].

In routine clinical practice, the magnitude and frequency of the swing in Δ*P*_es_ are probably sufficient to monitor respiratory effort.

### Transdiaphragmatic Pressure

A double balloon catheter can be used to monitor inspiratory swings in *P*_es_ and gastric pressure (*P*_ga_) to specifically quantify the pressure generated by the diaphragm (transdiaphragmatic pressure [*P*_di_]). During an inspiratory effort (depending on the pattern of thoracoabdominal motion), the diaphragm’s contractile effort moves the abdominal organs downwards, increasing abdominal pressure (positive swing in *P*_ga_) and expanding thoracic cavity (negative swing in *P*_es_). Even when thoracoabdominal motion is such that the diaphragm moves upward during inspiration (i.e., *P*_ga_ decreases), the contractile effort of the diaphragm is reflected by the fact that *P*_ga_ declines less than *P*_es_ (and thus *P*_di_ increases). This technique is used mainly in research rather than clinical practice.

## Monitoring Spontaneous Breathing by Occlusion Maneuvers

Expiratory and inspiratory occlusions represent easy, noninvasive, and reasonably reliable maneuvers to evaluate the safety of spontaneous breathing in assisted mechanical ventilation.

### Inspiratory Occlusion Maneuver

**Fig. 4:**
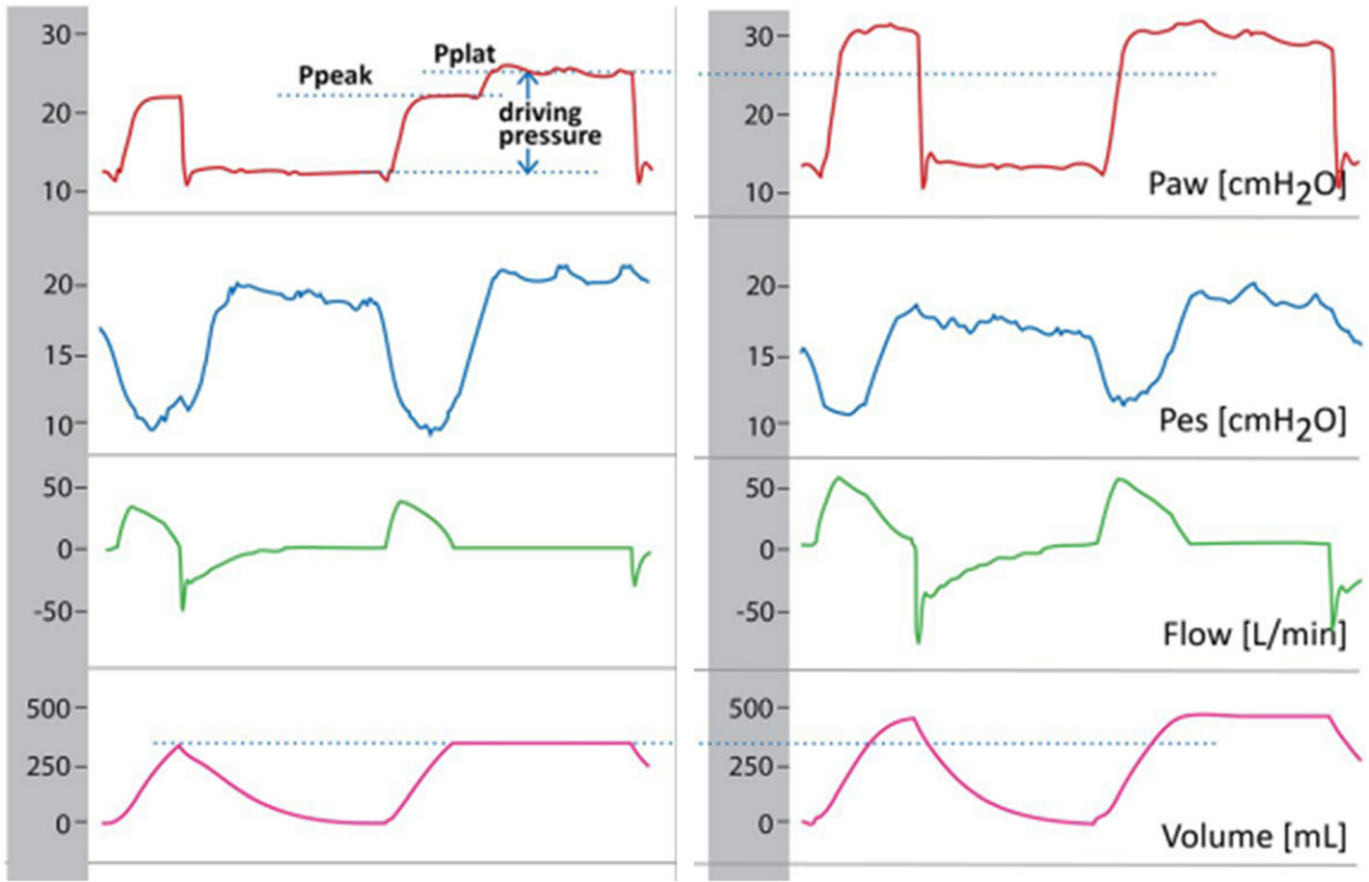
Measuring plateau pressure (*P*_plat_) during assisted mechanical ventilation (AMV). A brief inspiratory hold permits a reliable measure of *P*_plat_ in AMV, provided the patient relaxes with no immediate expiratory efforts. The difference between *P*_plat_ and positive end-expiratory pressure (PEEP) results in the driving pressure Δ*P*_aw_. In panel (**a**), the patient’s inspiratory effort is vigorous (greater esophageal swing): during inspiratory hold, the airflow stops and *P*_plat_ rises above *P*_peak_; the previous activated respiratory muscles relaxes and expires, causing *P*_aw_ to increase. In panel (**b**), the patient’s inspiratory effort is low: the difference between *P*_peak_ and *P*_plat_ is minimal, indicating minimal respiratory muscle effort during the current breath. This technique enables respiratory muscle activity to be assessed by measuring *P*_plat_. (Modified from [29] with permission)

Brief end-inspiratory occlusion maneuvers are widely used to measure plateau pressure (*P*_plat_) in passive mechanical ventilation. Driving pressure (Δ*P*), calculated as the difference between PEEP and *P*_pl_, reflects dynamic lung stress and lung injury risk and closely correlates to mortality in patients with ARDS [25]. Bellani et al. [29] suggested that a brief inspiratory occlusion maneuver can enable reliable measurements of *P*_plat_ even in assisted mechanical ventilation. During an inspiratory occlusion in assisted mechanical ventilation, patients relax the contracting inspiratory muscles at end-inspiration, resulting in an increase in Δ*P*_aw_, easily detectable on the ventilator waveform. When the patient is over-assisted and respiratory effort is low, *P*_aw_ drops during the occlusion (Fig. 4). A high *P*_plat_ and ΔP measured in this way raises concern for hyperdistention and lung injury. Bellani and colleagues [29] recently reported that Δ*P* and compliance measured by end-inspiratory occlusion maneuvers during assisted mechanical ventilation predict mortality, supporting the validity and relevance of these measures.

The measurement technique has some limitations. First, because the pressure is obtained under quasi-static conditions this measurement may underestimate the risk of regional lung injury due to the pendelluft mechanism of P-SILI [23]. Second, clinicians need to carefully evaluate the stability and pattern of the *P*_aw_ tracing during the occlusion to determine whether the measurement is confounded by the action of the abdominal muscles which may rapidly increase *P*_aw_ at the onset of neural expiration during the occlusion.

### Expiratory Occlusion Maneuver

Expiratory occlusions are ordinarily employed to measure intrinsic PEEP in passively ventilated patients or to measure maximal inspiratory pressure in spontaneously breathing patients during maximal volitional inspiratory efforts. However, the airway pressure swing during a brief, randomly applied end-expiratory occlusion maneuver (duration equal to one respiratory cycle) may actually be used to assess inspiratory effort. Under occluded conditions, the swing in airway pressure is exactly correlated to the swing in pleural pressure. Consequently, the airway pressure swing during the occlusion (Δ*P*_occ_) can be used to assess the presence and magnitude of pleural pressure swings due to patient respiratory effort (taking into account differences in pleural pressure swing between occluded and dynamic conditions). On this basis, Δ*P*_occ_ can be used to predict Δ*P*_es_, *P*_mus_, and Δ*P*_L_ during the respiratory cycle so long as the patient’s respiratory drive during the tidal breath is unchanged by a single, brief, and unexpected end-expiratory occlusion [30, 31]. A transient end-expiratory occlusion maneuver is a practical and noninvasive method to routinely detect insufficient or excessive respiratory effort and *P*_L_ during assisted mechanical ventilation [32, 33].

### Airway Occlusion Pressure

**Fig. 5:**
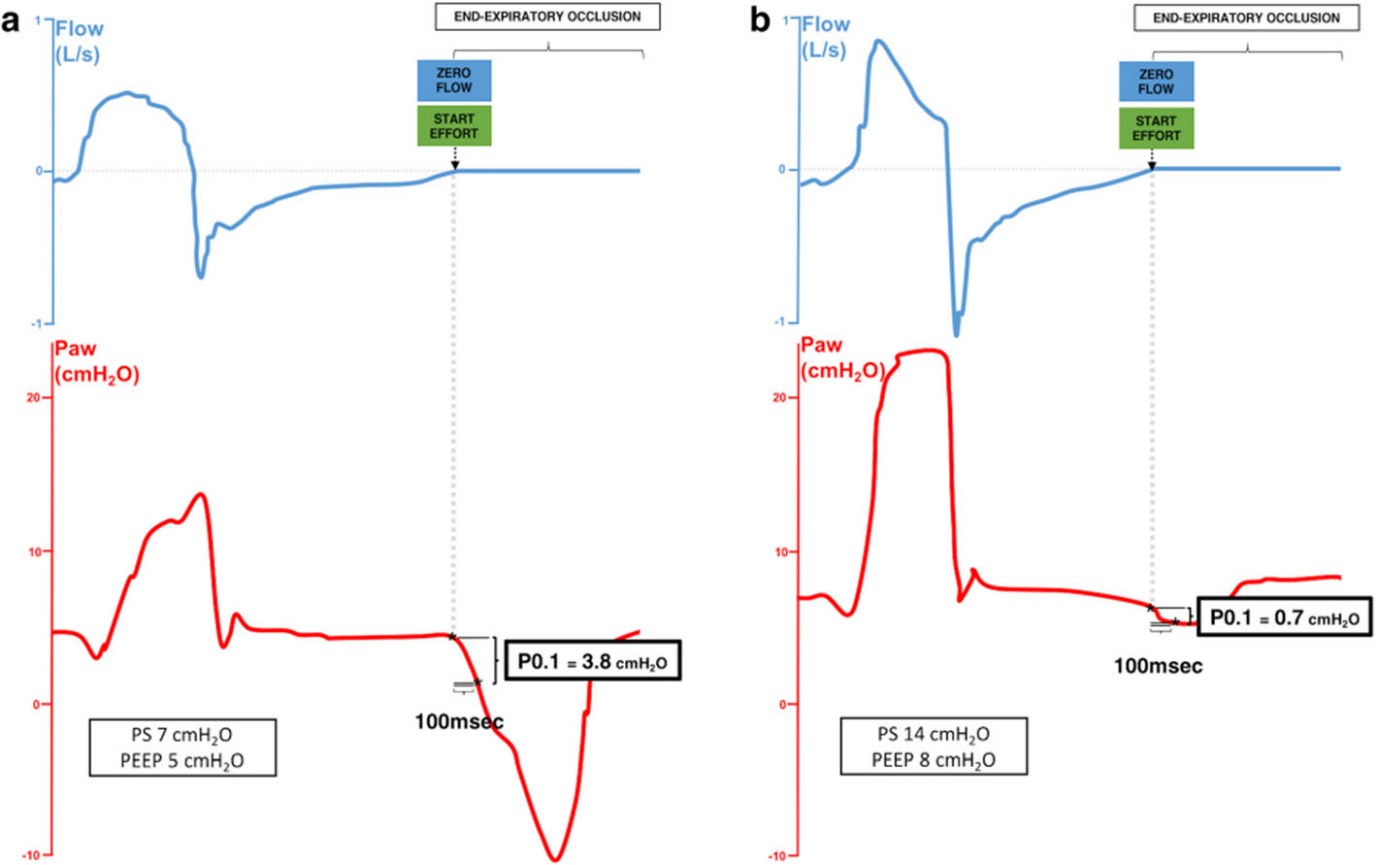
Airway occlusion pressure (*P*_0.1_) is the airway pressure (*P*_aw_) generated in the first 100 ms of inspiration against an expiratory occlusion. Importantly, the 100 ms time for *P*_0.1_ calculation should start at the point where the expiratory flow trace reaches zero (dashed line) to correct for potential intrinsic positive end-expiratory pressure (PEEP). *PS* pressure support level. (From [34] with permission)

The *P*_0.1_ (airway pressure generated in the first 100 ms of inspiration against an expiratory occlusion) provides a measure of the patient’s respiratory drive (Fig. 5) [34]. Whitelaw et al. [35] demonstrated that an occlusion does not modify cortical respiratory output until it is prolonged beyond 200 ms. Additionally, during the first 100 ms, respiratory pressure generation is independent of pulmonary mechanics or diaphragm function [35, 36]. Although the reliability of *P*_0.1_ has been confirmed only in small studies, a value between 1.5 and 3.5 cmH_2_O [37, 38] seems to be an easy method to guide clinicians to adjust ventilation during assisted mechanical ventilation [34, 39–41]. *P*_0.1_ values less than 1.5 cmH_2_O might suggest that respiratory effort is inadequate [42], and values greater than 3.5 cmH_2_O suggest high respiratory drive [37].

*P*_0.1_ has several advantages: it is easy and practical to obtain, and most modern ventilators have a function for measuring it. A method for setting the pressure support level based on the *P*_0.1_ value has been described [43]. *P*_0.1_ may have substantial intra-patient variability and several repeated measurements are required to estimate a stable mean value. Moreover, in hyperinflated patients, the intrinsic PEEP causes a delay in the fall in *P*_aw_, which might give rise to underestimation of *P*_0.1_. Conti et al. demonstrated that in this condition, commencing the 100 ms for the *P*_0.1_ measure when expiratory flow is equal to zero overcomes this problem [44].

## Monitoring Spontaneous Breathing by Diaphragm Electrical Activity

The use of a dedicated catheter fitted with electromyography electrodes permits continuous monitoring of the electrical activity of the diaphragm (EA_di_) [45]. EA_di_ has been demonstrated to be comparable to the transdiaphragmatic pressure, and is more practical than surface electromyography (EMG) [46].

When ventilation is driven by EA_di_ (during neurally adjusted ventilatory assist [NAVA]), patient-ventilator interaction improves [47, 48]; EA_di_ also helps clinicians to recognize different asynchronies [47, 49]. As demonstrated by Barwing et al. [50], the EA_di_ trend can be used to detect weaning failure at an early stage [51, 52]: it progressively increases in patients who ultimately fail their spontaneous breathing trial whereas diaphragm activity remains stable in patients who pass the trial. EA_di_ alterations appeared before signs of fatigue [50].

As an electrical signal, EA_di_ is an expression of respiratory motor output (the central nervous system activation of the diaphragm) and not of diaphragmatic force generation (effort). During resting breathing in healthy subjects, EA_di_ varies anywhere between 5 and 30 μV [53]. Because of this wide variation, it is difficult to specify a target EA_di_ to achieve during mechanical ventilation. Alternatively, EA_di_ can be used to estimate *P*_mus_ under different conditions of ventilator assistance [54]. By considering coupling between electrical activity and pressure generation constant during the time (neuro-mechanical coupling = *P*_mus_/EA_di_ obtained during expiratory occlusion), EA_di_ could permit a breath-by-breath assessment of *P*_mus_ during the normal breathing cycle.

## Monitoring Spontaneous Breathing by Diaphragm Ultrasound

The diaphragm ultrasound technique is noninvasive, easy to perform, and reproducible. Variation in diaphragm thickness during the respiratory cycle (thickening fraction, TF_di_) is correlated to respiratory pressure generation and EA_di_ [55] and can be used to detect diaphragm weakness [55]. TF_di_ values less than 30% during a maximal inspiratory effort detect diaphragm weakness with a high sensitivity [55]. Daily measurement of end-expiratory diaphragm thickness can detect structural changes in the respiratory muscles. In mechanically ventilated patients, a progressive increase in diaphragm thickness over time was correlated to excessive effort and may represent under-assistance myotrauma [3]. TF_di_ of 15–30% during tidal ventilation was associated with stable diaphragm thickness and the shortest duration of ventilation [4]. Ultrasound is best used for intermittent patient assessments, as it is not well suited for continuous monitoring.

## Conclusion

### Targets for Lung and Diaphragm-Protective Ventilation

Table 1 summarizes the different methods available to monitor inspiratory effort and respiratory drive in assisted mechanical ventilation, along with possible targets for safe spontaneous breathing as discussed throughout this chapter. The interpretation and application of measurements must always be guided by the clinical context. Different forms and phases of acute respiratory failure require somewhat different priorities: in early ARDS, close attention must be taken to avoid high inspiratory effort to limit VILI and P-SILI. Adjustments to ventilation and sedation to obtain a low level of inspiratory effort should be implemented as early as possible to avoid myotrauma.

**Table 1:** Potential target values for safe spontaneous breathing

Technique	Parameter	Possible target range of values for safe spontaneous breathing
Esophageal pressure	Peak end-inspiratory transpulmonary pressure (*P*_L_)	≤20 cmH_2_O
	Swing in transpulmonary pressure (Δ*P*_L_)	≤15 cmH_2_O
	Peak inspiratory muscle pressure (*P*_mus_)	5–10 cmH_2_O
	Esophageal pressure swing (Δ*P*_es_)	3–8 cmH_2_O
	Transdiaphragmatic pressure swing (Δ*P*_di_)	5–10 cmH_2_O
	Pressure time product (PTP)	50–100 cmH_2_O/s/min
Occlusion maneuvers	Inspiratory occlusion for plateau airway pressure (*P*_plat_)	≤30 cmH_2_O
	Inspiratory occlusion for driving pressure (Δ*P*_aw_ = *P*_plat_ − PEEP)	≤15 cmH_2_O
	Expiratory occlusion for estimated P_mus_	5–10 cmH_2_O
	Airway occlusion pressure (*P*_0.1_)	1.5–3.5 cmH_2_O
Electromyography	Diaphragm electrical activity (EA_di_)	Uncertain

*PEEP* positive end-expiratory pressure

It remains uncertain whether it is possible to achieve an acceptable level of respiratory effort during the acute phase of illness and this remains a key area for clinical investigation. For the present, clinicians should strive to be aware of patient respiratory effort and appreciate the potential benefits and harms of manipulating respiratory effort during acute respiratory failure.

## Data Availability

All data generated or analysed during this study are included in this published article.
